# CBT for Mild to Moderate Depression and Anxiety: A Guide to Low-Intensity Interventions

**DOI:** 10.1192/pb.bp.114.050476

**Published:** 2016-02

**Authors:** Chris O'Shea

This highly accessible book is pitched at healthcare professionals in primary and secondary care working with persons with anxiety and depression, with the aim of introducing and promoting the theory and practical applications of cognitive-behavioural therapy (CBT). This review coincided with my own supervised CBT case as part of my core training, and I found the book a helpful guide for practically applying cognitive and behavioural theoretical principles. Perhaps not surprisingly for a book on CBT, each chapter has clearly structured introductions, subheadings and summaries with recommended further reading. The contents are logically laid out, with succinct sections exploring initial assessment, problem identification, applying therapies to specific conditions and maintaining recovery. The subsections regarding session structure, goal-setting and maintenance models are especially useful, along with a most helpful chapter on applying the covered principles to difficulties with sleep. The book has a practical flavour throughout, with appropriate use of case vignettes, online materials to recommend to patients, and a wealth of resources within the appendices.

Reassuringly, the book is very clear that its main goal is not to act as a substitute for formal training or supervision, but rather to introduce the reader to the ideas and means to be more psychologically minded in one's practice. With this in mind, perhaps the main strength of the book is its awareness of its own limitations; it clearly signposts to further reading where appropriate, and emphasises the importance of supervision, and indeed how to optimise it.

In pitching to such a wide range of practitioners there may be some aspects of the book which some will find more relevant than others. Nonetheless, I feel the authors have produced a valuable guide to exploring the delivery of low-intensity CBT for depression/anxiety at a time when awareness of the importance of these therapies is increasing beyond psychiatric practice. Given this, I would recommend it as an introductory reference text.

